# Scaling Up Solutions to Toxic Contamination in Communities

**DOI:** 10.3390/ijerph16173034

**Published:** 2019-08-22

**Authors:** James W. Dearing

**Affiliations:** Department of Communication, Michigan State University, 404 Wilson Road, CAS 573A, East Lansing, MI 48824-1212, USA; dearjim@msu.edu; Tel.: 1-517-353-3259

**Keywords:** diffusion of innovations, research translation, community engagement, scale up

## Abstract

In this special issue of IJERPH, we feature studies conducted by research translation and community engagement teams that are funded through the Superfund Research Program in the United States. These and other teams funded by this program demonstrate how environmental and health communication research can contribute to generalizable lessons about helping and empowering contaminated communities. These types of applied behavioral, social and communication projects are important because while much about our communities is unique and must be addressed on a case by case basis, other aspects of research translation and community engagement processes are potentially generalizable across sites and can thus be used to scale up solutions to toxic contamination to other communities and countries more rapidly than would otherwise occur.

## 1. Introduction

Environmental and health communication have become increasingly important across the world, partly due to the increased number of communities, towns and cities badly polluted with toxic chemicals. Arsenic, lead, chlorinated contaminants like trichloroethylene, dioxins and polychlorinated biphenyls, mining trailings and slurries, solvents and oils and heavy metals that mix and seep into aquifers, and now per- and polyfluoralkyl substances (PFAS) that include thousands of compounds, all of these toxins threaten community ecosystems and human health. Community members fear not only for their family’s health, but also for the quality of their living environments as well as for their investments in homes, properties and the stock of liable companies in which their retirement savings may be vested. Governments at local, provincial or state, and national levels play central roles in identifying and characterizing risk and assigning responsibility, then overseeing remediation of polluted water, soil and air in varying degrees of partnership with community members themselves. At any particular site, the result is a complex interplay of stakeholder interests that co-occur while pollution is characterized and the site is litigated and cleaned up. 

Innovative and effective solutions to the problems of toxic contamination in communities commonly emanate from two paths: Change agencies, such as corporations, university labs and government agencies that are external to communities, and from the organizing of stakeholders within our communities. The former pathway is often termed *research translation* and concerns technical solutions as scientific results are moved via dissemination, technology transfer or other means to practical application. Organizing within a community is a pathway often referred to as *community engagement* and concerns social solutions as community stakeholders become involved in learning about contamination and remediation and in some cases collaborate in activities such as environmental monitoring, toxicology education and decision making. These pathways converge, for example, when university researchers seek community advice and involvement and when community members seek technical assistance from researchers outside of the community in question.

Unfortunately, the remediation of contaminated U.S. communities has not kept pace with the new identification of contaminated sites and the backlog of previously identified contaminated sites that remain unremediated. So despite the breadth and achievements of investments such as the Superfund Research Program, the systemic result is a widening *clean-up gap*. 

## 2. Narrowing the Clean-Up Gap

Effective social as well as technical innovations are necessary for the remediation of contaminated communities, but to make progress in narrowing the clean-up gap, we must additionally engage in the strategic sharing of what works across our communities in ways that will stimulate the diffusion of these innovations. Moreover, we must use design principles [1] from prior behavioral, social and communication research to accelerate the rate of innovation adoption and help community stakeholders to implement these solutions in ways that will both be effective and that will be compatible with community needs, norms and existing practices.

This is where the study of research translation approaches and community engagement processes becomes so important. They can provide answers to questions like:

- How is contamination understood by community members and what influences that understanding?
- What are the most effective ways of engaging and empowering community members in understanding and making decisions about their communities?
- Which pathways are most effective for reaching and affecting the technology-adoption decisions of state regulators, consulting engineers, and scientists in polluting companies? What are the attributes of technologies that explain the most variance in adoption (including licensing, purchasing and government-mandated technology adoption) decisions?
- Which ways of partnering with external stakeholder organizations prove to be most advantageous for the communities in question, and why?
- How is risk information best communicated to residents to result in safe behaviors?
- Do community concerns change over time and thus require dynamic approaches to research translation and community engagement?

Answers to questions such as these can then inform the development and testing of behavioral, social, and communication interventions that, with evidence of effect, can be scaled up and adapted to other communities and countries.

## 3. Conclusions

Investments by the U.S. National Institute of Environmental Health Sciences and the U.S. Environmental Protection Agency have led to impressive work in both studying and enacting research translation and community engagement as demonstrated at Superfund Research Program centers at the University of California San Diego, Columbia University, Boston University, the University of Puerto Rico, Duke University, Oregon State University and other grantees, as well as at the universities with work featured in this special issue of IJERPH. A next important stage is to use what we now know in designing for diffusion to other contaminated communities, whether the innovations to be scaled up are new applications of technologies such as the use of activated carbon to cleanse soil by attracting contaminants, or behavioral precautions such as the proper method of filleting locally caught fish to reduce the ingestion of toxins, or whether the featured innovations are evidence-based ways of engaging community members in learning and decision making.
